# Evaluation of the food grade expression systems NICE and pSIP for the production of 2,5-diketo-D-gluconic acid reductase from *Corynebacterium glutamicum*

**DOI:** 10.1186/2191-0855-3-7

**Published:** 2013-01-28

**Authors:** Vanja Kaswurm, Tien-Thanh Nguyen, Thomas Maischberger, Klaus D Kulbe, Herbert Michlmayr

**Affiliations:** 1 Food Biotechnology Laboratory, Department of Food Science and Technology, BOKU – University of Natural Resources and Life Sciences, Muthgasse 18, Vienna, 1190, Austria; 2 School of Biotechnology and Food Technology, Hanoi University of Science and Technology (HUST), Hanoi, Vietnam

**Keywords:** Ascorbic acid, 2,5-diketo-D-gluconic acid reductase, 2-keto-L-gulonic acid, *Corynebacterium glutamicum*, Food–grade, Lactic acid bacteria, pSIP, NICE

## Abstract

2,5-diketo-D-gluconic acid reductase (2,5-DKG reductase) catalyses the reduction of 2,5-diketo-D-gluconic acid (2,5-DKG) to 2-keto-L-gulonic acid (2-KLG), a direct precursor (lactone) of L-ascorbic acid (vitamin C). This reaction is an essential step in the biocatalytic production of the food supplement vitamin C from D-glucose or D-gluconic acid. As 2,5-DKG reductase is usually produced recombinantly, it is of interest to establish an efficient process for 2,5-DKG reductase production that also satisfies food safety requirements. In the present study, three recently described food grade variants of the *Lactobacillales* based expression systems pSIP (*Lactobacillus plantarum*) and NICE (*Lactococcus lactis*) were evaluated with regard to their effictiveness to produce 2,5-DKG reductase from *Corynebacterium glutamicum*. Our results indicate that both systems are suitable for 2,5-DKG reductase expression. Maximum production yields were obtained with *Lb. plantarum*/pSIP609 by pH control at 6.5. With 262 U per litre of broth, this represents the highest heterologous expression level so far reported for 2,5-DKG reductase from *C. glutamicum*. Accordingly, *Lb. plantarum/*pSIP609 might be an interesting alternative to *Escherichia coli* expression systems for industrial 2,5-DKG reductase production.

## Introduction

The bacterial enzyme 2,5-diketo-D-gluconic acid reductase (2,5-didehydrogluconate reductase; 2,5-DKG reductase; EC 1.1.1.274) is an NAD(P)(H)-dependent oxidoreductase assigned to the aldo-keto reductase (AKR) family ( Ellis 2002 ). 2,5-DKG reductase catalyses the stereo specific reduction of 2,5-diketo-D-gluconic acid (2,5-DKG) at position C-5 to 2-keto-L-gulonic acid (2-KLG), a key intermediate in the production of L-ascorbic acid (Anderson et al. 1985 ). At present, 2,5-DKG reductase is an integral part of several industrial processes designed to synthesize 2-KLG based on the 2,5-diketo-D-gluconic acid pathway (from D-glucose via D-gluconate, 2-keto-D-gluconate and 2,5-diketo-D-gluconate) ( Hancock and Viola 2002 , Bremus et al. 2006 ). An efficient hybrid process for the production of 2-KLG comprising the conversion of D-glucose or D-gluconic acid into 2,5-DKG by *Pectobacter cypripedii* HEPO1 (DSM 12939) and the subsequent reduction of 2,5-DKG to 2-KLG using 2,5-DKG reductase from *Corynebacterium glutamicum* was previously developed in our laboratory (Pacher et al. 2008 ). This process involves a commercially available glucose dehydrogenase in order to recycle the costly coenzyme NADPH in situ through oxidation of D-glucose to gluconic acid. An alternative biocatalytic process for 2-KLG production involving 2,5-DKG reductase has been presented by Genencor Inc. (Chotani et al. 2000 ).

The above mentioned processes depend on the heterologous (high-level) expression of the 2,5-DKG reductase gene (*dkr*), usually achieved with *Escherichia coli*. However, the utilization of genetically modified organisms (GMOs) to produce enzymes intended for food applications is strictly regulated (Pedersen et al. 2005 , Peterbauer et al. 2011 ). Lipopolysaccharide (endotoxin) production by *E. coli* is a further obstacle for protein expressions intended for food or medical purposes (Berczi et al. 1966 , Beutler and Rietschel 2003 ). Therefore, laborious and costly measures of down stream processing and quality control are required to comply with the purity and safety specifications for food grade enzymes, as recommended for example by the Joint FAO/WHO Expert Committee on Food Additives (JECFA) and the Food Chemical Codex (FCC). While recombinant (GMO) as well, an attractive alternative is to use expression hosts with the “generally recognized as safe” (GRAS) status, as defined by the US Food and Drug Administration (FDA). Although the reported performance of food grade expression systems is usually low compared to standard expression systems using *E. coli* (Nguyen et al. 2011a ), an advantage of applying GRAS (i.e., food-grade, Peterbauer et al. 2011 ) expression systems is that the costs to satisfy food safety requirements could be drastically reduced. Accordingly, efforts using lactic acid bacteria (LAB) as expression hosts have gained significance in the last decade (Peterbauer et al. 2011 ). Recently, examples of true food grade host/vector combinations have been presented and applied using the expression systems *Lactobacillus plantarum* / pSIP (Nguyen et al. 2011a ) and *Lactococcus lactis* / NICE (Maischberger et al. 2010 ). In both systems, antibiotic resistance marker genes have been replaced by selection markers (pSIP: *alr*, alanine racemase gene; NICE: *lacF*, gene encoding the soluble carrier enzyme IIA of the lactose specific phosphotransferase system) complementing corresponding gene deletions in the host chromosomes.

Previous studies on the above mentioned expression systems demonstrated high expression levels with bacterial β-galactosidase genes (Nguyen et al. 2011a , Maischberger et al. 2010 ). However, in these studies, the target genes originated from members of the same taxonomic order (*Lactobacillales*) as the expression hosts. It is therefore important as well to evaluate the performance of such LAB expression systems with genes of taxonomic distant origin. The aim of the present work was to evaluate the food grade expression systems pSIP and NICE for their capacity to produce the industrially important enzyme 2,5-DKG reductase from *C. glutamicum* (order *Actinomycetales*).

## Material and methods

### Materials

Chemicals for enzyme assays, protein analysis and media components were purchased from commercial suppliers at the highest available level of purity. 2,5-DKG, the substrate for 2,5-DKG reductase assays, was produced by fermentation of glucose with *Pectobacter cypripedii* (Pacher et al. 2008 ) and further purified as previously described (Kaswurm et al. 2012 ). All oligonucleotide primers used in this study are displayed in Table 1 and were synthesized by VBC-Biotech (Vienna, Austria). For preparation of genomic DNA from *C. glutamicum,* the Easy-DNA® Kit (Invitrogen, Carlsbad, CA) was used according to the instructions of the manufacturer. The PureYield™ Plasmid Miniprep System for isolation of *E. coli* plasmids and the Wizard® SV Gel & PCR Clean-UP kit for the purification of DNA fragments were obtained from Promega (Madison, WI, USA). The Quick Ligation Kit and restriction enzymes with their corresponding buffers were purchased from New England Biolabs (Ipswich, MA, USA). Phusion High-Fidelity PCR Master Mix (New England Biolabs) and a C1000 Thermal Cycler (Bio-Rad Laboratories Inc., Hercules, CA, USA) were used to amplify DNA by PCR.

**Table 1:** Oligonucleotide primers used for PCR amplifications in this study

**Primer name**	**Restriction enzyme**^**c**^	**Sequence (5′-3′)**	**Target gene**
V1^a^	-	ATGGATCAGAAGAATAAGCTTTC	*dkr*
V2^b^	*Spe*I	TCTACGACTAGTTCAGTTCAGATCATTCGG	*dkr*
V3^b^	*Xho*I	CTATCGCTCGAGTCAGTTCAGATCATTCGGG	*dkr*
P1^a^	*Spe*I	CGGAAATCACGGGAACTAGTCGCCAAA	P_*sppA*_, P_*sppQ*_
P2^b^	-	CGGTACCTACAACAGACATGGGAATCATACTCCTATATATTATT	P_*sppA*_
P3^b^	-	CGGTACCCACAACAGACATATATGCTGGCCAGCTAAGTA	P_*sppQ*_

^a^ forward primer.

^b^ reverse primer.

^c^ restriction sites are underlined.

### Molecular cloning

All bacterial strains/plasmids used in this study are shown in Table 2. Genomic DNA from *C. glutamicum* DSM 20301, cultivated in DSMZ medium 53 (German Collection of Microorganisms and Cell Cultures), was used as template for amplification of the 2,5-DKG reductase gene (*dkr*) (GenBank accession JQ407590.1). Plasmids were isolated from *Lactococcus* und *Lactobacillus* following the previously described protocol (O’ Sullivan and Klaenhammer 1993 ). All amplified sequences were verified by DNA sequencing (LGC Genomics, Berlin, Germany).

**Table 2:** **Bacterial strains and plasmids used in this study**^**a**^

**Strains or plasmids**	**Relevant characteristics**	**Reference or source**
**Strains**		
*Corynebacterium glutamicum*	DMSZ strain 20301	DMSZ
*Lactobacillus plantarum* WCFS1	a single colony isolated from *Lb. plantarum* NCIMB8826, which was originally isolated from human saliva (National Collection of Industrial and Marine Bacteria, Aberdeen, U.K.)	Kleerebezem et al. 2003
*Lactobacillus plantarum* TLG02	WCFS1 derivative, *Δalr*, D-alanine auxotroph, expression host	Nguyen et al. 2011a
*Lactococcus lactis* NZ3900	NZ3000 derivative, Δ*lacF*, *pepN*::*nisRK*, selection based on the ability to grow on lactose (*lacF*), expression host	de Ruyter et al. 1996
*Escherichia coli*		
MB2159	MC1000 derivative, D-alanine auxotroph, cloning host	Strych et al. 2001
NEB 5-alpha	cloning host	New England Biolabs
**Plasmids**		
pJet1.2/blunt	CloneJET™ PCR Cloning Kit	Fermentas
NICE derivative plasmids		
pNZ8150	*Cm*^*r*^, *P*_*nisA*_	Mierau and Kleerebezem 2005
pTM51R	*lac*F, pNZ8150 derivative containing *Lb. reuteri lacLM* genes downstream of P_*nisA*_	Maischberger et al. 2010
pVK51	*lac*F, pTM51R derivative containing the multiple cloning site (from pNZ8150) downstream of P_*nisA*_	this work
pVK51*dkr*	*lac*F, pVK51 derivative containing *C. glutamicum dkr* downstream of P_*nisA*_	this work
pSIP derived plasmids		
pSIP603R	*alr*, pSIP403 derivative containing *Lb. reuteri lacLM* controlled by P_*sppA*_	Nguyen et al. 2011a
pSIP609R	*alr*, pSIP409 derivative containing *Lb. reuteri lacLM* controlled by P_*sppQ*_	Nguyen et al. 2011a
pSIP603*dkr*	*alr*, pSIP603R derivative, *lacLM* replaced by *C. glutamicum dkr* controlled by P_*sppA*_	this work
pSIP609*dkr*	*alr*, pSIP609R derivative, *lacLM* replaced by *C. glutamicum dkr* controlled by P_*sppQ*_	this work

^a^*alr*: alanine racemase; *Cm*^*r*^: chloramphenicol resistance; *lac*F: the gene encoding the soluble carrier enzyme IIA from lactose specific PTS; *nisRK*: gene necessary for signal transduction integrated in the chromosome; P_*nisA*_: promoter nisin A; P_*sppA*_, P_*sppQ*_: the bacteriocin promoters in the *spp* gene cluster; *lacLM* beta-galactosidase encoding gene.

#### Construction of NICE-based expression vectors

**Figure 1:**
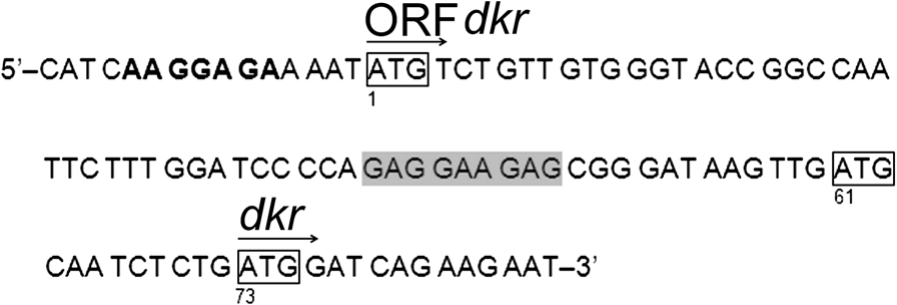
**N-terminal nucleotide sequence of the native *dkr *gene from *C. glutamicum *DSMZ 20301 (GenBank accession JQ407590.1).** A ribosomal binding site in the mRNA (bold print) is located upstream of the initiation codon (ATG, position 1). The second ATG codon in frame is located at position 61 and third ATG codon in frame at position 73. A region containing a high concentration of purine bases is highlighted.

NICE expression vectors were based on the pTM51 vector series as presented by Maischberger et al. ( 2010 ). The β-galactosidase encoding gene (*lacLM*) of pTM51R was excised with *Bgl*II and *Spe*I, and replaced by the multiple cloning site (mcs) from pNZ8150 ( Mierau and Kleerebezem 2005 ) using the same restriction sites; the resulting plasmid was designated pVK51. Primer pair V1/V2 (Table 1) was used to amplify the *dkr* gene from genomic *C. glutamicum* DNA. The PCR product was digested with *Spe*I, and the resulting fragment was ligated to plasmid pVK51 prepared by digestion with *Sca*I (blunt-end) and *Spe*I. This yielded the expression plasmid pVK51*dkr*. Additionally, the complete *dkr* open reading frame (ORF) with its start codon located 73 bases upstream of the *dkr* translation start (Figure 1), was cloned in vector pVK51 using 5′-ATGTCTGTTGTGGGTACCGG-3′ as foward and V2 (Table 1) as reverse primer. Both constructs were transformed into *L. lactis* NZ3900 (unable to grow on lactose), following the protocol of Holo and Nes (1989 ). Positive transformants were selected for their ability to grow on M17 medium ( Terzaghi and Sandine 1975 ) with agar (15 g L^-1^), supplemented with 1% lactose at 30°C.

#### Construction of pSIP-based expression vectors under control of P_*sppA*_ and P_*sppQ*_

The coding region of *dkr* was amplified with the primer pair V1/V3 (Table 1). Promoters P_*sppA*_ (pSIP603R) and P_*sppQ*_ (pSIP609R) (Nguyen et al. 2011a ), were amplified from the respective plasmid DNA using the primer pairs P1/P2 and P1/P3 (Table 1). The amplified *dkr* fragment was fused to the promoters P_*sppA*_ and P_*sppQ*_ by overlap extension polymerase chain reaction. Each of the two resulting fragments (P_*sppA*_:*dkr*, P_*sppQ*_:*dkr*) was ligated directly to the pJet1.2 blunt-end cloning vector (CloneJET PCR cloning kit; Fermentas GmbH, St. Leon-Rot, Germany) and transformed into chemically competent *E. coli* NEB 5-α cells (New England Biolabs). The inserts were excised with *Spe*I and *Xho*I (restriction sites on primers, Table 1) and ligated to a ~5.5 kb fragment obtained by cleavage of pSIP603R with the same restriction enzymes, resulting in the expression plasmids pSIP603*dkr* and pSIP609*dkr*. Following the same procedure, pSIP-based expression vectors containing the complete *dkr* ORF (designated pSIP603*dkr*ORF and pSIP609*dkr*ORF) were constructed. For *dkr* ORF amplification forward primer 5′-ATGTCTGTTGTGGGTACCGG-3′ and reverse primer V2 (Table 1) were used. After plasmid amplification with *E. coli* MB2159 (Strych et al. 2001 ), the constructs were electroporated into the D-alanine auxotroph expression host *Lb. plantarum* TLG02 (Nguyen et al. 2011a ) as described by Josson et al. ( 1989 ) and tranformants were cultivated in de Man, Rogosa and Sharpe broth (MRS medium; Oxoid, Basingstoke, U.K.) at 37°C without agitation. Competent cells of *E. coli* MB1259 were prepared and transformed according to the method of Inoue et al. ( 1990 ). Cultures of *E. coli* NEB 5-α and *E. coli* MB1259 transformants were grown in Luria-Bertani medium (LB; Sambrook et al. 1989 ) at 37°C with constant agitation (200 rpm). For the selection of *E. coli* NEB 5-α, ampicillin was added to a final concentration of 100 mg mL^-1^. For cultivation of *E. coli* MB2159 and *Lb. plantarum* TLG02 without plasmids, the respective growth media were supplemented with D-alanine (200 μg mL^-1^).

### Expression of 2,5-DKG reductase with food–grade vectors

Batch cultivations of LAB with food grade vectors were performed in computer-controlled stirred reactors (6 × 0.5 L) of the HT-Multifors system (Infors HT, Bottmingen, Switzerland). Comparative studies without and with pH control (pH 6.5) were performed. Culture pH was maintained by automated addition of sterile NaOH (1 M). To ensure homogenous distribution of the culture broth with limited oxygen transfer, a low agitation speed of 80 rpm was used. All experiments were performed in triplicate.

Inocula for the batch cultivations were prepared by transferring 20 μl of a frozen stock culture to 200 mL fresh medium (M17 for *L. lactis*; MRS for *Lb. plantarum*) and incubation at 30°C without shaking. After 12 hours, the cells were transferred to the bioreactor already containing the corresponding medium to reach an optical density at 600 nm (OD_600_) of ∼0.1. Expression was induced at an OD_600_ of 0.35 ± 0.03. For the induction of *Lb. plantarum* harbouring pSIP *alr*-based vectors, the synthetic peptide pheromone SppIP (Eijsink et al. 1996 ) (25 ng mL^-1^; CASLO Laboratory, Lyngby, Denmark) was used. To induce the NICE expression system with *L. lactis* NZ3900, nisin ( Mierau and Kleerebezem 2005 ), a 34 amino acid lantibiotic bacteriocin, was applied at a final concentration of 10 ng mL^-1^. In parallel to the induced cultures, noninduced negative controls were included to determine background activities and to calculate the induction factors (the quotient of specific activity obtained under induced conditions and the activity obtained under noninduced conditions). All experiments were carried out at 30°C for 20 hours following induction.

### Off-line analysis of parameters

Samples were taken in appropriate time intervals during the fermentations to monitor the growth of bacterial cultures by measuring OD_600_ and wet cell weight (WCW) after centrifugation at 15,000 × g for 15 min at 4°C. 2,5-DKG reductase activities and the total intracellular protein concentrations were determined in order to evaluate the expression levels. For that purpose, bacterial cells were harvested from 5 mL of culture by centrifugation at 3,220 × g for 10 min at 4°C, washed with Bis-Tris buffer (50 mM, pH 6.5) and resuspended in 500 μL of the same buffer. The cells were mechanically disrupted through bead beating with ∼1 g glass beads (average diameter of 0.5 mm) using a Precelly 24 glass bead mill (PEQLAB Biotechnologie GmbH, Erlangen, Germany). The cell-free crude extracts obtained after 10 min centrifugation at 9,000 × g (4°C) were used for 2,5-DKG reductase activity assays and determination of protein concentrations.

2,5-DKG reductase activity assay was performed spectrophotometrically as previously described (Kaswurm et al. 2012 ). One unit of 2,5-DKG reductase activity is defined as the enzyme quantity required to reduce 1 μmol of 2,5-DKG per min under assay conditions, which is equivalent to the production of 1 μmol of NADP^+^ per min (Kaswurm et al. 2012 ). Protein concentrations were determined by the dye binding method of Bradford (Bradford, 1976 ) using the Bio-Rad Protein Assay Kit (Bio-Rad Laboratories Inc.). Bovine serum albumin (BSA), in concentrations of 0.1 – 1.0 mg mL^-1^, was used for the standard calibration curve. All assays were performed in triplicate, and the data are expressed as mean values ± standard deviation (SD).

### Electrophoresis

SDS-PAGE was performed with a PerfectBlue standard vertical gel electrophoresis system (PEQLAB Biotechnologie GmbH) using 5% stacking gels and 10% separating gels. Samples were prepared according to method of Laemmli ( Laemmli 1970 ) and loaded in aliquots of 10 μL per line onto gel. Protein bands were stained using Coomassie blue R250. Precision Plus Protein™ Standard (Bio-Rad Laboratories Inc.) was used as molecular mass standard.

### Codon usage analysis

The fraction of usage of each codon of the *C. glutamicum dkr* gene by *L. lactis subsp. cremoris* MG1363 and *Lb. plantarum* WCFS1 (Kleerebezem et al. 2003 ), was predicted with the Graphical Codon Usage Analyser (Fuhrmann et al. 2004 ) and the results are presented as relative adaptiveness values. The codon usage table of *L. lactis subsp. cremoris* MG1363 is estimated based on 2572 CDS’s (739646 codons) and that of *Lb. plantarum* WCFS1 based on 3057 CDS’s (934462 codons) (Nakamura et al. 2000 ).

## Results

### Expression of the *C. glutamicum dkr* gene

**Figure 2:**
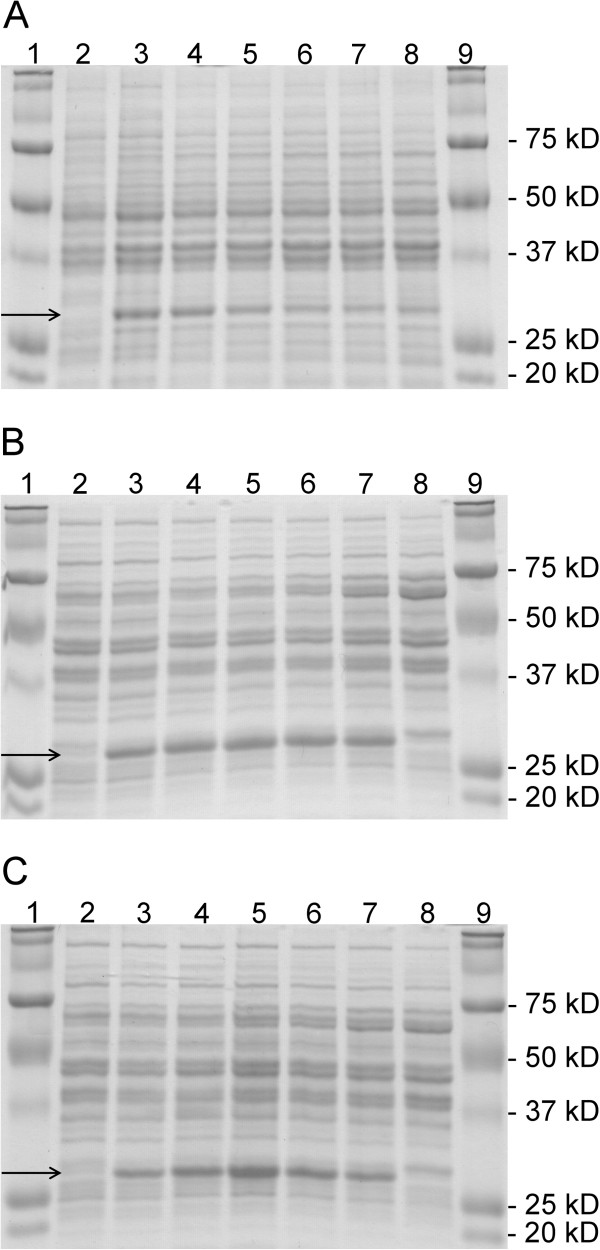
**SDS-PAGE of cell free extracts of strains *L. lactis *NZ3900, *Lb. plantarum *TLG02 and *Lb. plantarum *WCFS1 cultivated without pH maintainance.** Panel **A**: *L. lactis* NZ3900/pVK51*dkr*; Panel **B**: *Lb. plantarum*/pSIP603*dkr*; Panel **C**: *Lb. plantarum*/pSIP609*dkr*. Panel **A**: Lane 1 and Lane 9, molecular mass standard protein; Lane 2, culture uninduced; Lane 3-8, induced culture after 2, 4, 6, 8, 10 and 20 hours. Panel **B, C**: Lane 1 and Lane 9, molecular mass standard protein; Lane 2, culture uninduced; Lane 3-7, induced culture after 2, 4, 6, 8 and 10 hours; Lane 8, wild type *Lb. plantarum* WCFS1. The arrows indicate the band representing heterolougously expressed 2,5-DKG reductase.

**Figure 3:**
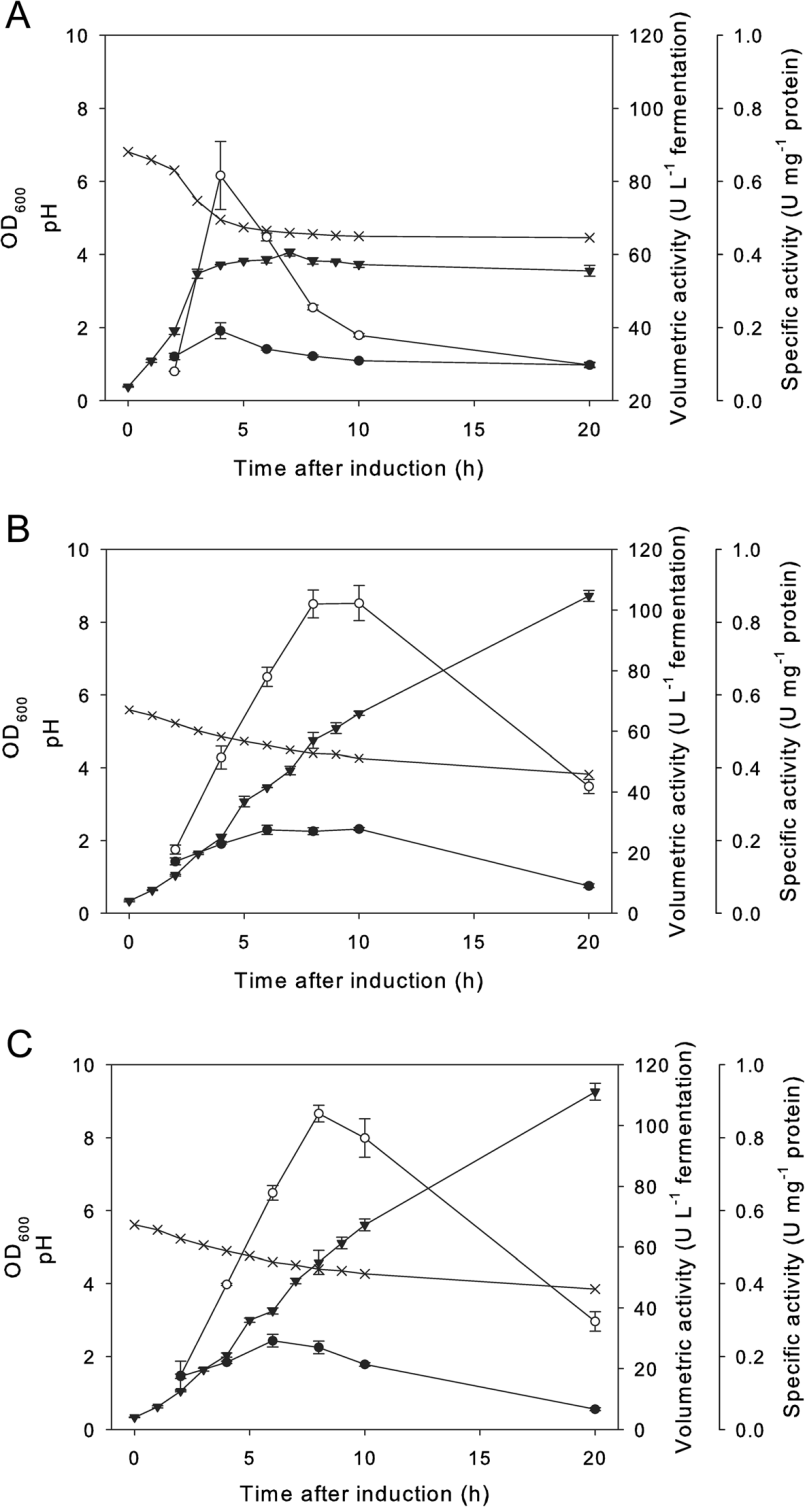
**Time course for growth of *L. lactis *NZ3900 or *Lb. plantarum *TLG02 cultivated without pH regulation.** Panel **A**: *L. lactis* NZ3900/pVK51*dkr*; Panel **B**: *Lb. plantarum*/pSIP603*dkr*; Panel **C**: *Lb. plantarum*/pSIP609*dkr*. The graph shows OD_600_ (triangles down), pH (crosshairs), volumetric 2,5-DKG reductase activity (units per liter of fermentation broth) (circles white) and specific activity (units per milligram protein) (circles black).

The first experiments were conducted without pH regulation during the fermentations. As judged by SDS-PAGE of the crude extracts (Figure 2), 2,5-DKG reductase could successfully be expressed with the three food–grade expression systems NICE, pSIP603 and pSIP609. Enzyme activities and protein concentrations were quantified during all experiments. The development of the monitored parameters over the fermentation time is plotted in Figure 3. In all cases, the highest yields of active 2,5-DKG reductase were observed during exponential growth. Table 3 summarizes the highest recorded activities during 2,5-DKG reductase expression. Consistent with previous reports (Maischberger et al. 2010 , Nguyen et al. 2011a ) the non-induced negative controls displayed some background activities, which were taken into account by calculating the net effect of induction (induction factor, Table 3).

**Table 3:** **Maximum 2,5-DKG reductase activities in cell free extracts of induced and noninduced *L. lactis *NZ3900 and *Lb. plantarum *TLG02 cultures**

**Not pH regulated cultivations of *L. lactis *and *Lb. plantarum***^**a**^					
**Strain /plasmid**	**Volumetric activity (U L**^**-1**^**fermentation broth)**		**Specific activity (U mg**^**-1**^**protein)**		**Induction factor**^**b**^
	**Induced**	**Noninduced**	**Induced**	**Noninduced**	
*L. lactis* NZ3900/pVK51*dkr*	81.6 ± 9.3	16.0 ± 0.24	0.191 ± 0.022	0.038 ± 0.001	5.0
*Lb. plantarum*/pSIP603*dkr*	102 ± 5.8	21.7 ± 2.0	0.232 ± 0.022	0.054 ± 0.002	4.3
*Lb. plantarum*/pSIP609*dkr*	104 ± 2.75	23.8 ± 2.2	0.243 ± 0.017	0.055 ±0.001	4.4
**pH regulated cultivations (pH 6.5) of *L. lactis *and *Lb. plantarum***^**a**^					
**Strain /plasmid**	**Volumetric activity (U L**^**-1**^** fermentation broth)**		**Specific activity (U mg**^**-1**^**protein)**		**Induction factor **^**b**^
	**Induced**	**Noninduced**	**Induced**	**Noninduced**	
*L. lactis* NZ3900/pVK51*dkr*	114 ± 1.9	14.6 ± 2.0	0.188 ± 0.001	0.022 ± 0.003	8.5
*Lb. plantarum*/pSIP603*dkr*	226 ± 5.9	27.2 ± 0.56	0.264 ± 0.026	0.032 ± 0.001	8.3
*Lb. plantarum*/pSIP609*dkr*	262 ± 1.7	30.2 ± 1.2	0.308 ± 0.016	0.033 ± 0.004	9.3

^a^ All data are mean values of three independent experiments. The maximum volumetric and specific activities measured in the culture are presented.

^b^ The induction factor was defined as the ratio between the specific activity obtained under induced conditions and the activity obtained under noninduced conditions.

With *L. lactis* NZ3900, the highest volumetric activities (82 U L^-1^ fermentation broth) were obtained 4 hours after induction. *L. lactis* reached a maximum OD_600_ of approx. 4 and a WCW of 3.6 g L^-1^. *Lb. plantarum* (with both pSIP603 and pSIP609) reached a maximum OD_600_ of just above 9 (WCW approx. 10 g L^-1^) and displayed maximum 2,5-DKG reductase activities of approximately 100 U L^-1^ after 8 hours of induction. The results of expressions without pH control (Figure 3) indicate that acid formation is the limiting factor for 2,5-DKG reductase production: In all cases, the volumetric activities of 2,5-DKG reductase decreased after reaching their maximum values, concomitant with a decrease of pH, levelling off at approximately pH 4. This is also evident on SDS-PAGE (Figure 2), as the intensities of the protein bands corresponding to 2,5-DKG reductase (31 kDa) decrease during continued fermentation.

**Figure 4:**
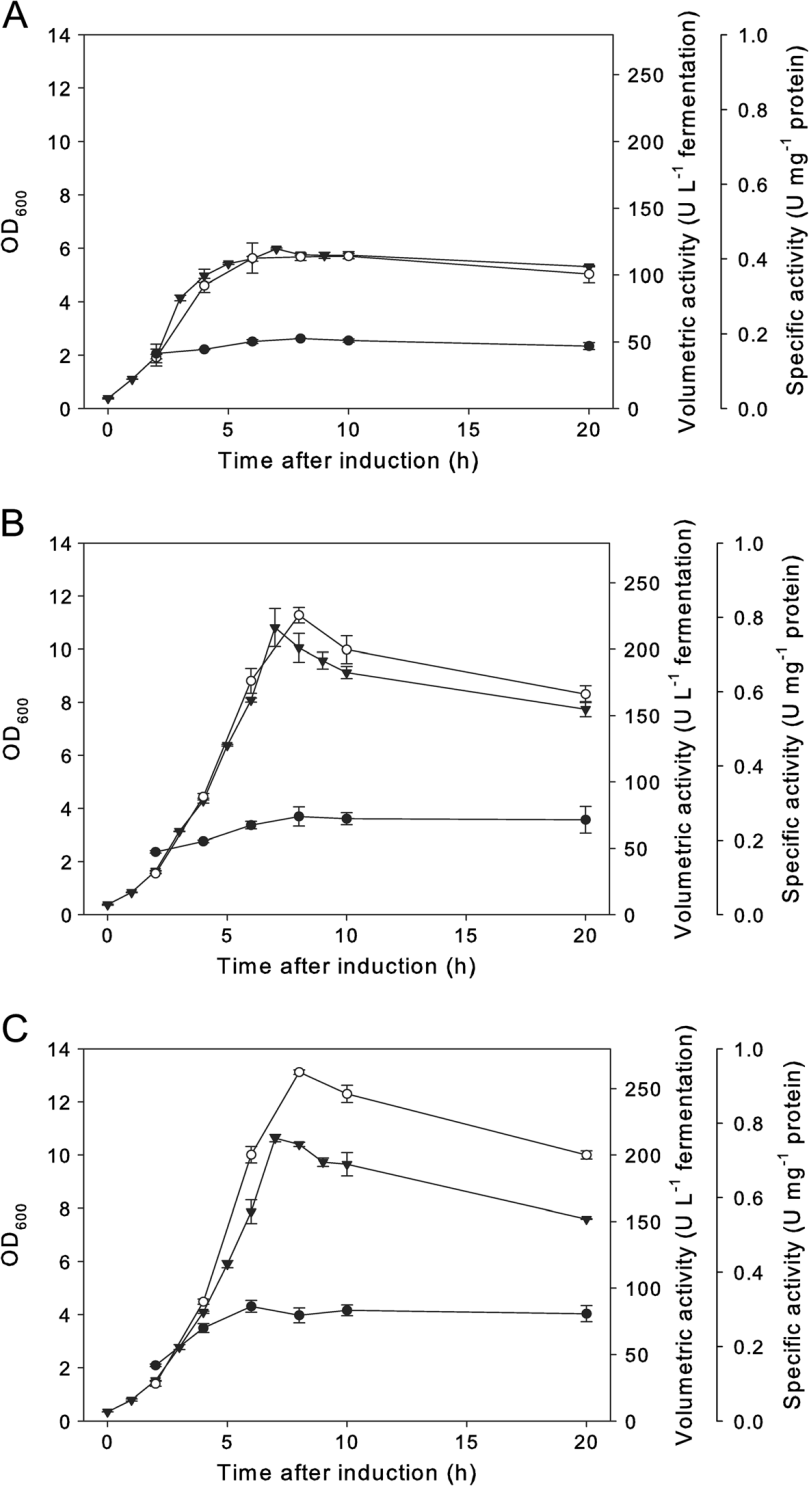
**Time course for growth of *L. lactis *NZ3900 or *Lb. plantarum *TLG02 cultivated with pH control at pH 6.5.** Panel **A**: *L. lactis* NZ3900/pVK51*dkr*; Panel **B**: *Lb. plantarum*/pSIP603*dkr*; Panel **C**: *Lb. plantarum*/pSIP609*dkr*. The graph shows OD_600_ (triangles down), volumetric 2,5-DKG reductase activity (units per liter of fermentation broth) (circles white) and specific activity (units per milligram protein) (circles black).

The results of *dkr* gene expression with pH control at 6.5, but otherwise identical conditions are plotted in Figure 4. Compared to the experiments without pH control, the volumetric activities of recombinant 2,5-DKG reductase could be increased by factors ranging from 1.4 (*L. lactis* NZ3900) to 2.5 (*Lb. plantarum*/pSIP609) (see Table 3). pH control resulted in higher cell densities as well: *L. lactis* reached a maximum OD_600_ of approx. 6 (WCW of 5.5 g L^-1^) and *Lb. plantarum* an OD_600_ of approx. 10 (WCW = 11.7 g L^-1^ with pSIP603 and 11.3 g L^-1^ with pSIP609) after 7 – 8 hours of induction. After reaching the growth maximum, volumetric activities began to drop in the *Lb. plantarum* cultures (Figure 4B, C). In the studies using the *L. lactis* expression system, volumetric acitivites remained rather stable over the recorded fermentation period. In all cases, pH-regulated cultivation resulted in increased stability of the recombinant 2,5-DKG reductase as monitored during 20 hours of induced fermentations (compare Figures 3 and 4).

The highest production levels of 2,5-DKG reductase were obtained with the system *Lb. plantarum*/pSIP609, resulting in 104 U L^-1^ without pH regulation and 262 U L^-1^ with pH control at 6.5. Although formation of recombinant 2,5-DKG reductase by *Lb. plantarum* (both pSIP603 and pSIP609) was higher than with *L. lactis*, the induction factors did not differ significantly because of slightly higher basal expression of noninduced *Lb. plantarum* TLG02 cells. It can be concluded that some basal 2,5-DKG reductase expression, caused by “leakage” of the corresponding promoters, occured in noninduced *Lb. plantarum* TLG02 cells (Table 3). Additional experiments using wild type *Lb. plantarum* WCFS1 (ancestral strain of TLG02, see Table 2) (Kleerebezem et al. 2003 ) were performed in MRS medium under equal conditions as described above, but without induction. The highest 2,5-DKG reductase activities detected were 11.8 ± 0.8 U L^-1^ without pH regulation and 13.6 ± 0.9 U L^-1^ with pH control at 6.5. Database research using the BLASTp algorithm (NCBI Database; http://www.ncbi.nlm.nih.gov/; Altschul et al. 1997 ) revealed the presence of several putative oxidoreductases in the *Lb. plantarum* WCFS1 genome with up to 47% amino acid sequence identities with *dkr.* This circumstance might be an explanation for the recorded 2,5-DKG reductase background activities as well. Putative aldo/keto reductases with up to 48% amino acid sequence identities to *dkr* could also be identified in the published genome of *L. lactis* MG1363 (ancestral strain of *L. lactis* NZ3900) (de Ruyter et al. 1996 ).

### Investigation of an alternative "*dkr gene* variant"

In this and previous studies (Kaswurm et al. 2012 , Pacher 2006 ) the *dkr* gene was cloned and expressed such that the third in-frame ATG codon of the complete open reading frame (ORF) (GenBank accession JQ407590.1) was used as translation start (Figure 1). This is a consequence of previous experiments conducted in our laboratory that demonstrated the presence of two protein bands with distinct electrophoretic mobilities (both identified as *dkr* gene products by MALDI-TOF analysis) when the complete *dkr* ORF (His_6_-tagged) was expressed with an *E. coli* expression system ( Pacher 2006 ). Sequence analysis of the *dkr* gene shows a region with a high concentration of purine bases (GAG GAA GAG), located downstream of the first initiation codon (between position 36 and 54, see Figure 1), which may have been recognized as an alternative ribosomal binding site by *E. coli*. Subsequent experiments (*E. coli)* using the third ATG codon as translational start (Figure 1) resulted in the presence of only a single discernable band on SDS-PAGE and especially, higher expression yields than with the complete ORF. Interestingly, the automated gene product annotations (i.e., predicted start of translation) of the currently available coding sequences of the *dkr* gene from *C. glutamicum* (GenBank, 99% sequence identities to JQ407590.1; BLASTn; Altschul et al. 1997 ), differ in the above discussed respect, whereas either the first, second or third ATG codon are predicted as putative translation start sites (accession nos. CAF21024.1.; BAF55246.1; CCH25497.1 and BAB99752.1).

Following these considerations, the expression of the complete *dkr* ORF was investigated with all presented LAB systems/variants as well (see results in Additional file 1, Additional file 2, Additional file 3, Additional file 4). Interestingly, in contrast to *E. coli*, only a single protein band was visible on SDS-PAGE (see Additional file 1). However, the yields of 2,5-DKG reductase activities (in terms of both volumetric and specific activities) achieved by expression of the complete *dkr* ORF were significantly lower than by expression of the "*dkr* gene" (starting at the third ATG codon in frame) with all systems (see Additional file 2, Additional file 3, Additional file 4).

A possible explanation for the improved expression charateristics of "*dkr"* compared to the complete ORF may be indicated by codon usage analysis: Compared to *L. lactis subsp. cremoris* MG1363 and *Lb. plantarum* WCFS1, the mean difference of the codon usage in ORF of *dkr* gene from *C. glutamicum* was 39.6% and 36.7% for the complete ORF and 40.1% and 37.1% for *dkr* gene, respectively. Additionally, according to the codon usage table of *L. lactis subsp. cremoris* MG1363 (Additional file 5) an analysis of usage of the first 50 codons of the complete ORF and *dkr* gene, shows that 10 codons (ORF) and 8 codons (*dkr*), respectively can be considered “rare codons” (i.e. codons used in less than 20% of the cases) or “very rare codons” (i.e. codons used in less than 10% of the cases). Conversely, for *Lb. plantarum* there is no rare codon with a low fraction of usage within the first 50 codons of both ORF and *dkr* gene from *C. glutamicum* (Additional file 6).

## Discussion

The majority of the so far published studies concerned with the heterologous expression of *dkr* genes (*Corynebacterium* sp.) were focussed on 2,5-DKG reductase optimization by site-directed mutagenesis and the kinetic characterisation of the obtained mutants after expression in *E. coli*, rather than the optimization of expression yields (Banta et al. 2002a, b , Powers 1996 , Sanli et al. 2004 , Banta and Anderson 2002 ). However, *Erwinia* species (*Erwinia herbicola* and *Erwinia citreus*) that naturally accumulate 2,5-DKG from D-glucose have been used as expression host for *dkr* as well, and have been employed in the one-step production of 2-KLG (Anderson et al. 1985 , Grindley et al. 1988 , Wührer 2006 ). The expression degree of *dkr* in *Erwinia* strains was evaluated through the production titer of 2-KLG, and the highest productivity rate of 6.6 g L^-1^ d^-1^ was achieved with *Erwinia citreus*, mutant strain ER1026 (Grindley et al. 1988 ).

The focus of the present study was to determine the value of two recently developed LAB based food-grade expression systems for the production of 2,5-DKG reductase. The best results (judged by enzyme activity in the crude extract) were obtained with *Lb. plantarum*/pSIP609. Interestingly, the corresponding production yields were in the same range as those previously obtained by *dkr* expression with *E. coli*/pET21d (approx. 200 U L^-1^ fermentation broth) (Kaswurm et al. 2012 ). Additionally, this is the highest expression level so far reported for this enzyme and shows that LAB systems are suitable for *dkr* expression as well. However, it needs to be critically discussed whether LAB systems could compete with *E. coli* in an industrial production process. Considering the current costs of the required growth media (at the time of writing: MCHGly medium approx. 3 € per liter; MRS medium approx. 9 €), the estimated costs for 2,5-DKG reductase production with *Lb. plantarum* would be at least 3 fold compared to *E. coli*. A strong argument to employ food grade expression systems however is that such, the costs to satisfy food safety requirements may be significantly reduced (Mierau et al. 2005 ). Although the options presented here do not represent "self-clones" and have therefore to be considered as GMO, the use of gram positive expression hosts is still highly attractive because lipopolysaccharide formation can be avoided such, which might indeed reduce the costs for downstream processing and quality assurance required for "food grade" enzymes. In addition, the here applied food grade expression systems do not contain potentially harmful, transferable antibiotic resistence markers (Peterbauer et al., 2011 ). Since vitamin C is an important and widely used food supplement, expression of 2,5-DKG reductase with such food grade systems could indeed represent an interesting option.

In this regard, it is important to note that research on LAB expression systems is still in progress, and it can reasonable be expected that expression efficiencies of such systems will be much improved over the next years. An important aspect to improve a particular system is the choice of the inducible promotor, which was also indicated in the present study: Heterologous expression levels (Table 3) of the *C. glutamicum dkr* gene with *Lb. plantarum* (pSIP603, pSIP609), clearly indicate that the expression characteristics of the same system can be significantly influenced by the used promotor (P_*sppA*_ and P_*sppQ*_, respectively), as pSIP609 showed improved expression levels compared to pSIP603 in all cases. These data stand in contrast to the results recently published by Nguyen and co-workers (Nguyen et al. 2011a ), who found no significant differences between pSIP603 and pSIP609 comparing the levels of β-galactosidase expressions. However, our results are in excellent accordance with those obtained for the β-glucuronidase (GusA) from *E. coli* and aminopeptidase N (PepN) from *L. lactis* expressed with *Lb. plantarum* NC8 harbouring corresponding pSIP based vectors with erythromycin resistance (Sørvig et al. 2005 ).

Further strategies recently discussed involve the increase of plasmid copy numbers and optimization of mRNA secondary structure in the translational initiation region (TIR) (Nguyen et al. 2011b , Friehs 2004 , Ganoza and Louis 1994 ). Another important aspect is to analyse the codon usage preference among organisms used as expression systems. Accordingly, by modification of the target gene towards the set of codons that the host organism (*L. lactis*. or *Lb. plantarum*) naturally uses in its highly expressed genes, the risk of tRNA depletion during translation can be minimized and hence the heterologous expression by lactic acid bacteria could be further optimized ( Fuglsang 2003 ). In addition, design of fermentation medium and further optimization of cultivation conditions using a well reasoned strategy ( Kennedy and Krouse 1999 , Berlec et al. 2008 ) could contribute to multiple increases of cell densities and expression productivities.

In conclusion, with *dkr* from *C. glutamicum* as example, our results confirm that LAB expression systems such as NICE and pSIP are indeed attractive candidates for high level protein production and may gain further interest for industrial purposes in the near future.

## Abbreviations

AKR: Aldo-keto reductase; Alr: Alanine racemase gene; Cm^r^: Chloramphenicol resistance; 2,5-DKG reductase: 2,5-diketo-D-gluconic acid reductase; 2,5-DKG: 2,5-diketo-D-gluconic acid; FDA: Food and Drug Administration; GMO: Genetically modified organism; GusA: β-glucuronidase; 2-KLG: 2-keto-L-gulonic acid; LAB: Lactic acid bacteria; *lacF*: The soluble carrier enzyme IIA encoding gene; *lacLM*: Overlapping genes encoding β-galactosidase; NADPH: Nicotinamide adenine dinucleotide phosphate (reduced form); NADP^+^: Nicotinamide adenine dinucleotide phosphate (oxidized form); NICE: Nisin controlled gene expression; ORF: Open reading frame; PepN: Aminopeptidase N; P_*nisA*_: Promoter nisin A; P_*sppA*_ P_*sppQ*_: The bacteriocin promoters in the *spp* gene cluster.

## Competing interest

The authors declare that they have no competing interests.

## Supplementary Material

Additional file 1 Figure S1 SDS-PAGE of cell free extracts of strains *L. lactis* NZ3900, *Lb. plantarum* TLG02 and *Lb. plantarum* WCFS1 cultivated without pH maintainance. Panel A: *L. lactis* NZ3900/pVK51ORF*dkr*; Panel B: *Lb. plantarum*/pSIP603ORF*dkr*; Panel C: *Lb. plantarum*/pSIP609ORF*dkr*. Panel A: Lane 1 and Lane 8, molecular mass standard protein; Lane 2, culture uninduced; Lane 3-7, induced culture after 2, 4, 6, 8 and 10 hours. Panel B, C: Lane 1 and Lane 8, molecular mass standard protein; Lane 2, culture uninduced; Lane 3-6, induced culture after 2, 4, 6 and 8 hours; Lane 7, wild type *Lb. plantarum* WCFS1. The arrows indicate the band representing heterolougously expressed complete *dkr* ORF. Click here for file

Additional file 2 Table S1 Maximum activities of 2,5-DKG reductase measured in cell free extracts of induced and noninduced *L. lactis* and *Lb. plantarum* TLG02 after expression of the complete *dkr* ORF. Click here for file

Additional file 3 Figure S2 Time course for growth of *L. lactis* NZ3900 or *Lb. plantarum* TLG02 cultivated without pH regulation. Panel A: *L. lactis* NZ3900/pVK51ORF*dkr*; Panel B: *Lb. plantarum*/pSIP603ORF*dkr*; Panel C: *Lb. plantarum*/pSIP609ORF*dkr*. The graph shows OD_600_ (triangles down), pH (crosshairs), 2,5-DKG reductase activity (units per liter of fermentation broth) (circles white) and specific activity (units per milligram protein) (circles black). Click here for file

Additional file 4 Figure S3 Time course for growth of *L. lactis* NZ3900 or *Lb. plantarum* TLG02 cultivated with pH control at pH 6.5. Panel A: *L. lactis* NZ3900/pVK51ORF*dkr*; Panel B: *Lb. plantarum*/pSIP603ORF*dkr*; Panel C: *Lb. plantarum*/pSIP609ORF*dkr*. The graph shows OD_600_ (triangles down), 2,5-DKG reductase activity (units per liter of fermentation broth) (circles white) and specific activity (units per milligram protein) (circles black). Click here for file

Additional file 5 Figure S4 Codon usage analysis of the 50 first codons in complete *dkr* ORF (A) and *dkr* (B) of *C. glutamicum* in *L. lactis subsp. cremoris* MG1363. The vertical axis indicates the relative adaptiveness values (%) of triplet codons in *L. lactis subsp. cremoris* MG1363. The codons used in less than 20% of the cases are considered as rare and their codon usage fraction appears in grey. Click here for file

Additional file 6 Figure S5 Codon usage analysis of the 50 first codons in complete *dkr* ORF (A) and *dkr* (B) of *C. glutamicum* in *Lb. plantarum* WCFS1. The vertical axis indicates the relative adaptiveness values (%) of triplet codons in *Lb. plantarum* WCFS1. The codons used in less than 20% of the cases are considered as rare and their codon usage fraction appears in grey. Click here for file

## References

[B1] Altschul SF Madden TL Schäffer AA Zhang J Zhang Z Miller W Lipman DJ Gapped BLAST and PSI-BLAST: a new generation of protein database search programs Nucleic Acids Res 1997 25 3389 3402 10.1093/nar/25.17.3389 9254694 PMC146917

[B2] Anderson S Marks CB Lazarus R Miller J Stafford K Seymour J Light D Rastetter W Estell D Production of 2-keto-L-gulonate, an intermediate in L-ascorbate synthesis, by a genetically modified Erwinia herbicola Science 1985 230 144 149 10.1126/science.230.4722.144 17842676

[B3] Banta S Anderson S Verification of a novel NADH-binding motif: combinatorial mutagenesis of three amino acids in the cofactor-binding pocket of Corynebacterium 2,5-diketo-D-gluconic acid reductase J Mol Evol 2002 55 623 631 10.1007/s00239-002-2345-x 12486521

[B4] Banta S Swanson BA Wu S Jarnagin A Anderson S Alteration of the specificity of the cofactor-binding pocket of Corynebacterium 2,5-diketo-D-gluconic acid reductase A Protein Eng 2002 15 131 140 10.1093/protein/15.2.131 11917149

[B5] Banta S Swanson BA Wu S Jarnagin A Anderson S Optimizing an artificial metabolic pathway: engineering the cofactor specificity of Corynebacterium 2,5-diketo-D-gluconic acid reductase for use in vitamin C biosynthesis Biochemistry 2002 41 6226 6236 10.1021/bi015987b 12009883

[B6] Berczi I Bertók L Bereznai T Comparative Studies on the Toxicity of Escherichia coli Lipopolysaccaride Endotoxin in Various Animal Species Can J Microbiol 1966 12 1070 1071 10.1139/m66-143 5339644

[B7] Berlec A Tompa G Slapar N Fonović UP Rogelj I Štrukelj B Optimization of fermentation conditions for the expression of sweet-tasting protein brazzein in Lactococcus lactis Lett Appl Microbiol 2008 46 227 231 10.1111/j.1472-765X.2007.02297.x 18215220

[B8] Beutler B Rietschel ET Timeline: Innate immune sensing and its roots: the story of endotoxin Nat Rev Immunol 2003 3 169 176 10.1038/nri1004 12563300

[B9] Bradford MM A rapid and sensitive method for the quantitation of microgram quantities of protein utilizing the principle of protein-dye binding Anal Biochem 1976 72 248 254 10.1016/0003-2697(76)90527-3 942051

[B10] Bremus C Herrmann U Bringer-Meyer S Sahm H The use of microorganisms in L-ascorbic acid production J Biotechnol 2006 124 196 205 10.1016/j.jbiotec.2006.01.010 16516325

[B11] Chotani G Dodge T Hsu A Kumar M LaDuca R Trimbur D Weyler W Sanford K The commercial production of chemicals using pathway engineering Biochim Biophys Acta Protein Struct Mol Enzymol 2000 1543 434 455 10.1016/S0167-4838(00)00234-X 11150618

[B12] de Ruyter PG Kuipers OP Beerthuyzen MM van Alen-Boerrigter I de Vos WM Functional analysis of promoters in the nisin gene cluster of Lactococcus lactis J Bacteriol 1996 178 3434 3439 8655538 10.1128/jb.178.12.3434-3439.1996 PMC178110

[B13] Eijsink VG Brurberg MB Middelhoven PH Nes IF Induction of bacteriocin production in Lactobacillus sake by a secreted peptide J Bacteriol 1996 178 2232 2237 8636023 10.1128/jb.178.8.2232-2237.1996 PMC177930

[B14] Ellis EM Microbial aldo-keto reductases FEMS Microbiol Lett 2002 216 123 131 10.1111/j.1574-6968.2002.tb11425.x 12435492

[B15] Friehs K Plasmid copy number and plasmid stability Adv Biochem Eng Biotechnol 2004 86 47 82 15088763 10.1007/b12440

[B16] Fuglsang A Lactic acid bacteria as prime candidates for codon optimization Biochem Biophys Res Commun 2003 312 285 291 10.1016/j.bbrc.2003.10.120 14637134

[B17] Fuhrmann M Hausherr A Ferbitz L Schödl T Heitzer M Hegemann P Monitoring dynamic expression of nuclear genes in Chlamydomonas reinhardtii by using a synthetic luciferase reporter gene Plant Mol Biol 2004 55 869 881 15604722 10.1007/s11103-004-2150-6

[B18] Ganoza MC Louis BG Potential secondary structure at the translational start domain of eukaryotic and prokaryotic mRNAs Biochimie 1994 76 428 439 10.1016/0300-9084(94)90120-1 7849110

[B19] Grindley JF Payton MA van de Pol H Hardy KG Conversion of glucose to 2-keto-L-gulonate, an intermediate in L-ascorbate synthesis, by a recombinant strain of Erwinia citreus Appl Environ Microbiol 1988 54 1770 1775 16347687 10.1128/aem.54.7.1770-1775.1988 PMC202744

[B20] Hancock RD Viola R Biotechnological approaches for L-ascorbic acid production Trends Biotechnol 2002 20 299 305 10.1016/S0167-7799(02)01991-1 12062975

[B21] Holo H Nes IF High-frequency transformation, by electroporation, of Lactococcus lactis subsp. cremoris grown with glycine in osmotically stabilized media Appl Environ Microbiol 1989 55 3119 3123 16348073 10.1128/aem.55.12.3119-3123.1989 PMC203233

[B22] Inoue H Nojima H Okayama H High efficiency transformation of Escherichia coli with plasmids Gene 1990 96 23 28 10.1016/0378-1119(90)90336-P 2265755

[B23] Josson K Scheirlinck T Michiels F Platteeuw C Stanssens P Joos H Dhaese P Zabeau M Mahillon J Characterization of a gram-positive broad-host-range plasmid isolated from Lactobacillus hilgardii Plasmid 1989 21 9 20 10.1016/0147-619X(89)90082-6 2727147

[B24] Kaswurm V Pacher C Kulbe KD Ludwig R 2,5-Diketo-gluconic acid Reductase from Corynebacterium glutamicum: characterization of stability, catalytic properties and inhibition mechanism for use in vitamin C synthesis Process Biochem 2012 47 2012 2019 10.1016/j.procbio.2012.07.014

[B25] Kennedy M Krouse D Strategies for improving fermentation medium performance: a review J Ind Microbiol Biotechnol 1999 23 456 475 10.1038/sj.jim.2900755

[B26] Kleerebezem M Boekhorst J van Kranenburg R Molenaar D Kuipers OP Leer R Tarchini R Peters SA Sandbrink HM Fiers MWEJ Stiekema W Lankhorst RMK Bron PA Hoffer SM Groot MNN Kerkhoven R de Vries M Ursing B de Vos WM Siezen RJ Complete genome sequence of Lactobacillus plantarum WCFS1 Proc Natl Acad Sci 2003 100 1990 1995 10.1073/pnas.0337704100 12566566 PMC149946

[B27] Laemmli UK Cleavage of structural proteins during the assembly of the head of bacteriophage T4 Nature 1970 227 680 685 10.1038/227680a0 5432063

[B28] Maischberger T Mierau I Peterbauer CK Hugenholtz J Haltrich D High-level expression of Lactobacillus β-galactosidases in Lactococcus lactis using the food-grade, nisin-controlled expression system NICE J Agric Food Chem 2010 58 2279 2287 10.1021/jf902895g 20092320

[B29] Mierau I Kleerebezem M 10 years of the nisin-controlled gene expression system (NICE) in Lactococcus lactis Appl Microbiol Biotechnol 2005 68 705 717 10.1007/s00253-005-0107-6 16088349

[B30] Mierau I Leij P van Swam I Blommestein B Floris E Mond J Smid EJ Industrial-scale production and purification of a heterologous protein in Lactococcus lactis using the nisin-controlled gene expression system NICE: the case of lysostaphin Microb Cell Fact 2005 4 15 10.1186/1475-2859-4-15 15921518 PMC1173137

[B31] Nakamura Y Gojobori T Ikemura T Codon usage tabulated from international DNA sequence databases: status for the year 2000 Nucleic Acids Res 2000 28 292 292 10.1093/nar/28.1.292 10592250 PMC102460

[B32] Nguyen TT Mathiesen G Fredriksen L Kittl R Nguyen TH Eijsink VGH Haltrich D Peterbauer CK A food-grade system for inducible gene expression in Lactobacillus plantarum using an alanine racemase-encoding selection marker J Agric Food Chem 2011 59 5617 5624 10.1021/jf104755r 21504147

[B33] Nguyen TT Nguyen TH Maischberger T Schmelzer P Mathiesen G Eijsink V Haltrich D Peterbauer C Quantitative transcript analysis of the inducible expression system pSIP: comparison of the overexpression of Lactobacillus spp. β-galactosidases in Lactobacillus plantarum Microb Cell Fact 2011 10 46 56 10.1186/1475-2859-10-46 21696579 PMC3155831

[B34] O’Sullivan DJ Klaenhammer TR Rapid mini-prep isolation of high-quality plasmid DNA from Lactococcus and Lactobacillus spp Appl Environ Microbiol 1993 59 2730 2733 16349028 10.1128/aem.59.8.2730-2733.1993 PMC182348

[B35] Pacher C Recombinant DKR from Corynebacterium glutamicum: screening, cloning, purification and use of the enzyme for L-ascorbic acid production 2006 Dissertation, University of Natural Resources and Life Sciences, Vienna

[B36] Pacher C Kulbe KD Steiner E Rembart G Method for producing ascorbic acid using Pectobacter cypripedii. WIPO Patent WO/2008/144792A1 4 Dez 2008

[B37] Pedersen MB Iversen SL Sørensen KI Johansen E The long and winding road from the research laboratory to industrial applications of lactic acid bacteria FEMS Microbiol Rev 2005 29 611 624 10.1016/j.fmrre.2005.04.001 15935510

[B38] Peterbauer C Maischberger T Haltrich D Food-grade gene expression in lactic acid bacteria Biotechnol J 2011 6 1147 1161 10.1002/biot.201100034 21858927

[B39] Powers DB Structure/function studies of 2,5-Diketo-D-gluconic acid reductases 1996 Dissertation, University of Medicine and Dentistry of New Jersey, Piscataway, NJ

[B40] Sambrook J Fritsch EF Maniatis T Molecular cloning: a laboratory manual 1989 2 N.Y Cold Spring Harbor Laboratory Press, Cold Spring Harbor

[B41] Sanli G Banta S Anderson S Blaber M Structural alteration of cofactor specificity in Corynebacterium 2,5-diketo-D-gluconic acid reductase Protein Sci 2004 13 504 512 10.1110/ps.03450704 14718658 PMC2286697

[B42] Sørvig E Mathiesen G Naterstad K Eijsink VG Axelsson L High-level, inducible gene expression in Lactobacillus sakei and Lactobacillus plantarum using versatile expression vectors Microbiology 2005 151 2439 2449 10.1099/mic.0.28084-0 16000734

[B43] Strych U Penland RL Jimenez M Krause KL Benedik MJ Characterization of the alanine racemases from two Mycobacteria FEMS Microbiol Lett 2001 196 93 98 10.1111/j.1574-6968.2001.tb10547.x 11267762

[B44] Terzaghi BE Sandine WE Improved medium for lactic streptococci and their bacteriophages Appl Microbiol 1975 29 807 813 16350018 10.1128/am.29.6.807-813.1975 PMC187084

[B45] Wührer F Erwinia (Pectobacter) cypripedii as Reichstein intermediates producing cell factory 2006 Dissertation, University of Natural Resources and Life Sciences, Vienna

