# Supplementary material for: Evaluation of the food grade expression systems NICE and pSIP for the production of 2,5-diketo-D-gluconic acid reductase from Corynebacterium glutamicum
Source: AMB Express. 2013 Jan 28;3:7. doi: 10.1186/2191-0855-3-7 (PMC3565945; doi:10.1186/2191-0855-3-7)
Supplement: Additional file 1 Figure S1 — SDS-PAGE of cell free extracts of strains L. lactis NZ3900, Lb. plantarum TLG02 and Lb. plantarum WCFS1 cultivated without pH maintainance. Panel A: L. lactis NZ3900/pVK51ORFdkr; Panel B: Lb. plantarum/pSIP603ORFdkr; Panel C: Lb. plantarum/pSIP609ORFdkr. Panel A: Lane 1 and Lane 8, molecular mass standard protein; Lane 2, culture uninduced; Lane 3-7, induced culture after 2, 4, 6, 8 and 10 hours. Panel B, C: Lane 1 and Lane 8, molecular mass standard protein; Lane 2, culture uninduced; Lane 3-6, induced culture after 2, 4, 6 and 8 hours; Lane 7, wild type Lb. plantarum WCFS1. The arrows indicate the band representing heterolougously expressed complete dkr ORF. [file 2191-0855-3-7-S1.pdf]

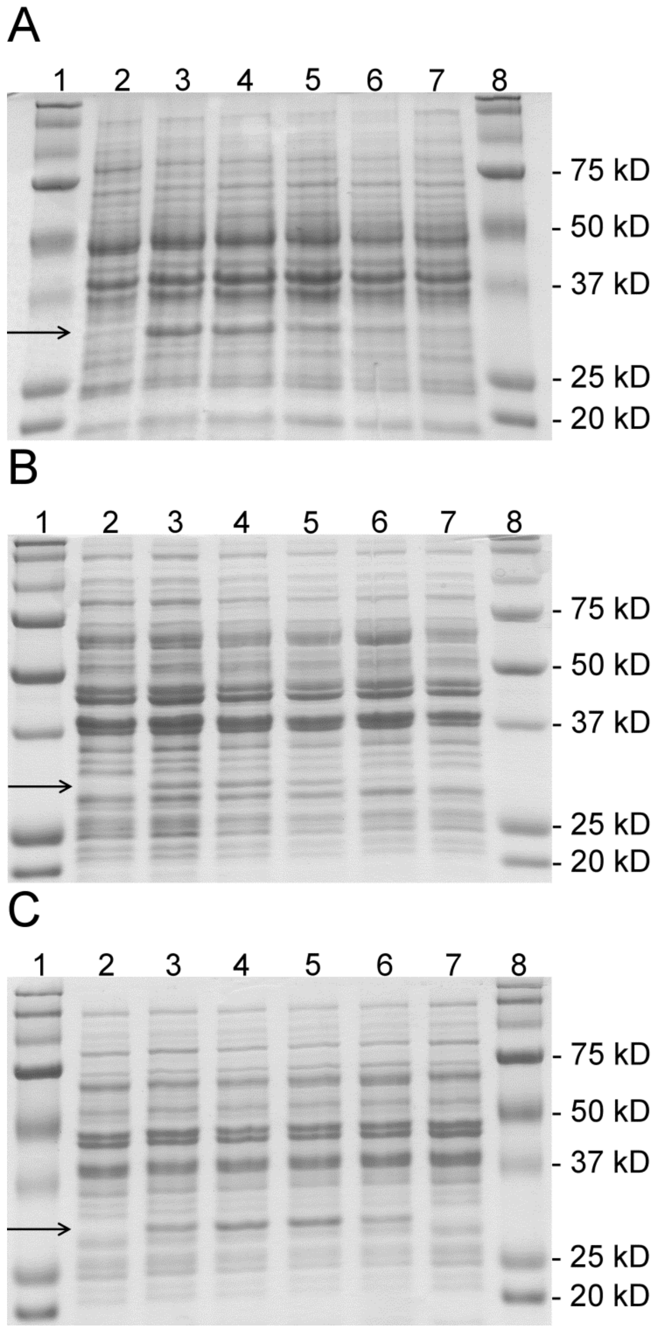

Supplementary figure 1 SDS-PAGE of cell free extracts of strains *L. lactis* NZ3900, *Lb. plantarum* TLG02 and *Lb. plantarum* WCFS1 cultivated without pH maintainance.

Panel A: *L. lactis* NZ3900/pVK51ORF*dkr*; Panel B: *Lb. plantarum*/pSIP603ORF*dkr*; Panel C: *Lb. plantarum*/pSIP609ORF*dkr*. Panel A: Lane 1 and Lane 8, molecular mass standard protein; Lane 2, culture uninduced; Lane 3-7, induced culture after 2, 4, 6, 8 and 10 hours. Panel B, C: Lane 1 and Lane 8, molecular mass standard protein; Lane 2, culture uninduced; Lane 3-6, induced culture after 2, 4, 6 and 8 hours; Lane 7, wild type *Lb. plantarum* WCFS1.

The arrows indicate the band representing heterolougously expressed complete *dkr* ORF .
