# Supplementary material for: Evaluation of the food grade expression systems NICE and pSIP for the production of 2,5-diketo-D-gluconic acid reductase from Corynebacterium glutamicum
Source: AMB Express. 2013 Jan 28;3:7. doi: 10.1186/2191-0855-3-7 (PMC3565945; doi:10.1186/2191-0855-3-7)
Supplement: Additional file 2 Table S1 — Maximum activities of 2,5-DKG reductase measured in cell free extracts of induced and noninduced L. lactis and Lb. plantarum TLG02 after expression of the complete dkr ORF. [file 2191-0855-3-7-S2.pdf]

Supplementary table 1 Maximum activities of 2,5-DKG reductase measured in cell free extracts of induced and noninduced *L. lactis* and *Lb. plantarum* TLG02 after expression of the complete *dkr* ORF

not pH regulated cultivations of *L. lactis* and *Lb. plantarum* [a]					
strain /plasmid	volumetric activity (U L^{-1} fermentation broth)		specific activity (U mg^{-1} protein)		Induction factor [b]
	induced	noninduced	induced	noninduced	
L. lactis NZ3900/pVK51ORF*dkr*	44.8 ± 0.58	14.1 ± 1.5	0.111 ± 0.009	0.032 ± 0.002	3.7
Lb. plantarum/pSIP603ORF*dkr*	43.2 ± 1.8	13.1 ± 0.17	0.114 ± 0.001	0.046 ± 0.003	2.5
Lb. plantarum/pSIP609ORF*dkr*	47.1 ± 1.6	15.7 ± 0.37	0.152 ± 0.004	0.049 ± 0.001	3.1

pH regulated cultivations (pH 6.5) of *L. lactis* and *Lb. plantarum* [a]					
strain /plasmid	volumetric activity (U L^{-1} fermentation broth)		specific activity (U mg^{-1} protein)		Induction factor [b]
	induced	noninduced	induced	noninduced	
L. lactis NZ3900/pVK51ORF*dkr*	70.4 ± 7.9	13.8 ± 1.3	0.144 ± 0.009	0.026 ± 0.002	5.5
Lb. plantarum/pSIP603ORF*dkr*	63.4 ± 0.87	14.4 ± 1.7	0.095 ± 0.002	0.020 ± 0.004	4.8
Lb. plantarum/pSIP609ORF*dkr*	107 ± 9.3	20.0 ± 0.19	0.168 ± 0.017	0.032 ± 0.002	5.3

[a] All data are mean values of three independent experiments. The maximum volumetric and specific activities measured in the culture are presented.

[b] The induction factor was defined as the ratio between the specific activity obtained under induced conditions and the activity obtained under noninduced conditions
