# Supplementary material for: Evaluation of the food grade expression systems NICE and pSIP for the production of 2,5-diketo-D-gluconic acid reductase from Corynebacterium glutamicum
Source: AMB Express. 2013 Jan 28;3:7. doi: 10.1186/2191-0855-3-7 (PMC3565945; doi:10.1186/2191-0855-3-7)
Supplement: Additional file 3 Figure S2 — Time course for growth of L. lactis NZ3900 or Lb. plantarum TLG02 cultivated without pH regulation. Panel A: L. lactis NZ3900/pVK51ORFdkr; Panel B: Lb. plantarum/pSIP603ORFdkr; Panel C: Lb. plantarum/pSIP609ORFdkr. The graph shows OD600 (triangles down), pH (crosshairs), 2,5-DKG reductase activity (units per liter of fermentation broth) (circles white) and specific activity (units per milligram protein) (circles black). [file 2191-0855-3-7-S3.pdf]

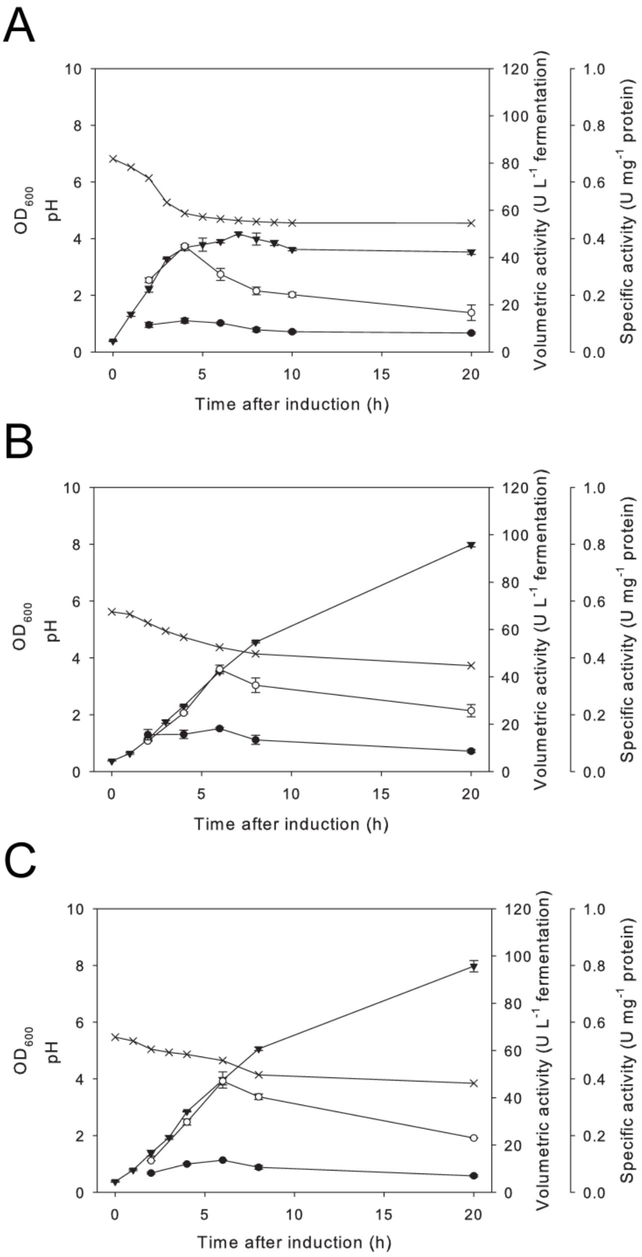

Supplementary figure 2 Time course for growth of *L. lactis* NZ3900 or *Lb. plantarum* TLG02 cultivated without pH regulation.

Panel A: *L. lactis* NZ3900/pVK51ORF*dkr*; Panel B: *Lb. plantarum*/pSIP603ORF*dkr*; Panel C: *Lb. plantarum*/pSIP609ORF*dkr*. The graph shows OD_{600} (triangles down), pH (crosshairs), 2,5-DKG reductase activity (units per liter of fermentation broth) (circles white) and specific activity (units per milligram protein) (circles black).
