# Supplementary material for: Evaluation of the food grade expression systems NICE and pSIP for the production of 2,5-diketo-D-gluconic acid reductase from Corynebacterium glutamicum
Source: AMB Express. 2013 Jan 28;3:7. doi: 10.1186/2191-0855-3-7 (PMC3565945; doi:10.1186/2191-0855-3-7)
Supplement: Additional file 6 Figure S5 — Codon usage analysis of the 50 first codons in complete dkr ORF (A) and dkr (B) of C. glutamicum in Lb. plantarum WCFS1. The vertical axis indicates the relative adaptiveness values (%) of triplet codons in Lb. plantarum WCFS1. The codons used in less than 20% of the cases are considered as rare and their codon usage fraction appears in grey. [file 2191-0855-3-7-S6.pdf]

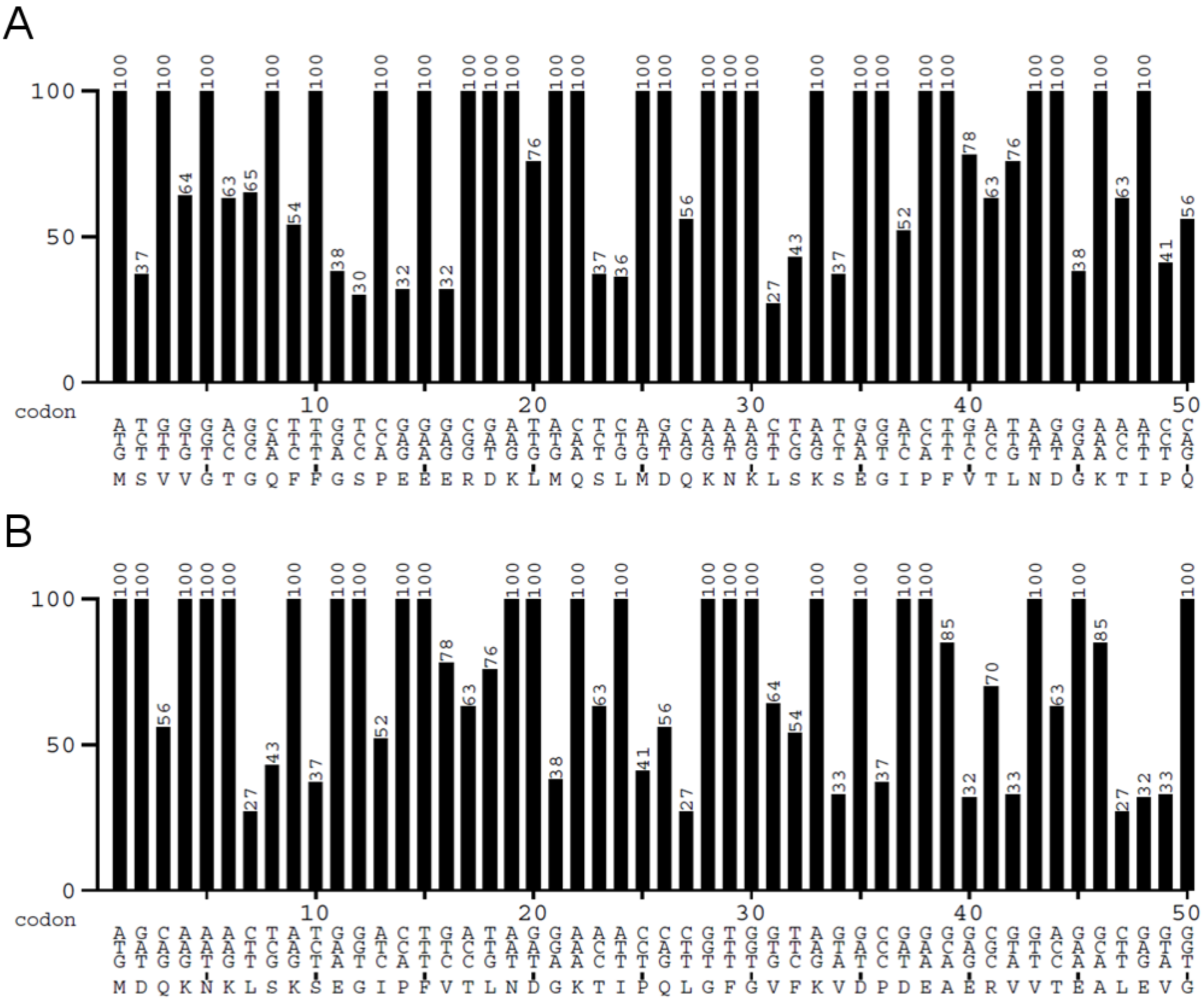

Supplementary figure 5 Codon usage analysis of the 50 first codons in complete *dkr* ORF (A) and *dkr* (B) of *C. glutamicum* in *Lb. plantarum* WCFS1.

The vertical axis indicates the relative adaptiveness values (%) of triplet codons in *Lb. plantarum* WCFS1. The codons used in less than 20% of the cases are considered as rare and their codon usage fraction appears in grey.
